# Supplementary material for: Time trends in social contacts of individuals according to comorbidity and vaccination status, before and during the COVID-19 pandemic
Source: BMC Med. 2022 May 23;20:199. doi: 10.1186/s12916-022-02398-x (PMC9126104; doi:10.1186/s12916-022-02398-x)
Supplement: Supplementary file 1 — Additional file 1: Table S1. Distribution of comorbidity status among confirmed cases in Quebec adults. Table S2. STROBE Statement—Checklist of items that should be included in reports of cross-sectional studies. Table S3. Classifications of active physical comorbidities. Table S4. Sample size calculations. Table S5. Key socio-demographic characteristics of participants, by period. Table S6. Time trends in the mean number of social contacts of individuals with and without active physical comorbidities. Table S7. Time trends in the mean total number of social contacts of individuals with and without active physical comorbidities at risk of COVID-19 complications. Table S8. Vaccination coverage with one dose of individuals with and without active physical comorbidities in the third wave. Table S9. Recommended Quebec priority groups for vaccination against COVID-19. Table S10. Mean total number of social contacts in the third wave of individuals with and without active physical comorbidities according to vaccination status with one dose. Figure S1. Distribution of participants according to classifications of comorbidities. Figure S2. Time trends in vaccination coverage with one dose of individuals with and without active physical comorbidities in the third wave. Example of questions S1. Questions on health conditions. Example of questions S2. Example of the social contact diary. [file 12916_2022_2398_MOESM1_ESM.pdf]

ADDITIONAL FILE 1

Table of contents

Table S1. Distribution of comorbidity status among confirmed cases in Quebec adults 2

Table S2. STROBE Statement—Checklist of items that should be included in reports of cross-sectional studies .3

Table S3. Classifications of active physical comorbidities 5

Table S4. Sample size calculations..6

Table S5. Key socio-demographic characteristics of participants, by period..7

Table S6. Time trends in the mean number of social contacts of individuals with and without active physical comorbidities 9

Table S7. Time trends in the mean total number of social contacts of individuals with and without active physical comorbidities at risk of COVID-19 complications 12

Table S8. Vaccination coverage with one dose of individuals with and without active physical comorbidities in the third wave 14

Table S9. Recommended Quebec priority groups for vaccination against COVID-19...15

Table S10. Mean total number of social contacts in the third wave of individuals with and without active physical comorbidities according to vaccination status with one dose 16

Figure S1. Distribution of participants according to classifications of comorbidities...17

Figure S2. Time trends in vaccination coverage with one dose of individuals with and without active physical comorbidities in the third wave 18

Example of questions S1. Questions on health conditions 19

Example of questions S2. Example of the social contact diary 21

Table S1. Distribution of comorbidity status among confirmed cases in Quebec adults*

	1st wave		2nd wave		3rd wave	
	n	%	n	%	n	%
**All adults**						
Without comorbidities	19265	51.2	94879	56.6	29710	62.3
With comorbidities	18331	48.8	72634	43.4	17997	37.7
**18-45 years old**						
Without comorbidities	12529	71.0	64201	73.6	20491	75.3
With comorbidities	5119	29.0	23064	26.4	6715	24.7
**46-65 years old**						
Without comorbidities	6169	47.3	27159	50.3	8303	52.9
With comorbidities	6877	52.7	26846	49.7	7386	47.1
**66-75 years old**						
Without comorbidities	416	17.9	2609	22.9	722	25.3
With comorbidities	1914	82.1	8787	77.1	2128	74.7
**>75 years old**						
Without comorbidities	151	3.3	910	6.1	194	9.9
With comorbidities	4421	96.7	13937	93.9	1768	90.1

1st wave: February 23rd to July 11th 2020; **2nd wave:** August 23rd 2020 to March 20th 2021; **3rd wave:** March 21st to July 13th 2021.
Methodology used to generate data from the first wave is available online [6]. The same methodology has been used for the other waves.
* Cases from nursing homes are excluded

Table S2. STROBE Statement—Checklist of items that should be included in reports of *cross-sectional studies* [30]

	Item No	Recommendation	Page No
**Title and abstract**	1	(*a*) Indicate the study's design with a commonly used term in the title or the abstract	2
		(*b*) Provide in the abstract an informative and balanced summary of what was done and what was found	2, 3
**Introduction**			
Background/rationale	2	Explain the scientific background and rationale for the investigation being reported	4, 5
Objectives	3	State specific objectives, including any prespecified hypotheses	5, 6
**Methods**			
Study design	4	Present key elements of study design early in the paper	6
Setting	5	Describe the setting, locations, and relevant dates, including periods of recruitment, exposure, follow-up, and data collection	6, 7, 8, 9<br>Figure 1
Participants	6	(*a*) Give the eligibility criteria, and the sources and methods of selection of participants	6
Variables	7	Clearly define all outcomes, exposures, predictors, potential confounders, and effect modifiers. Give diagnostic criteria, if applicable	7, 8, 9, 10
Data sources/ measurement	8*	For each variable of interest, give sources of data and details of methods of assessment (measurement). Describe comparability of assessment methods if there is more than one group	7, 8, 9
Bias	9	Describe any efforts to address potential sources of bias	9, 10
Study size	10	Explain how the study size was arrived at	10
Quantitative variables	11	Explain how quantitative variables were handled in the analyses. If applicable, describe which groupings were chosen and why	9, 10
Statistical methods	12	(*a*) Describe all statistical methods, including those used to control for confounding	9, 10
		(*b*) Describe any methods used to examine subgroups and interactions	9, 10
		(*c*) Explain how missing data were addressed	9
		(*d*) If applicable, describe analytical methods taking account of sampling strategy	N/A
		(*e*) Describe any sensitivity analyses	10<br>Appendix p.5
**Results**			
Participants	13*	(a) Report numbers of individuals at each stage of study—eg numbers potentially eligible, examined for eligibility, confirmed eligible, included in the study, completing follow-up, and analysed	10<br>Appendix p.17
		(b) Give reasons for non-participation at each stage	10

	Item No	Recommendation	Page No
		(c) Consider use of a flow diagram	N/A
Descriptive data	14*	(a) Give characteristics of study participants (eg demographic, clinical, social) and information on exposures and potential confounders	10, 11 Table 1 Appendix p.7, 8, 17
		(b) Indicate number of participants with missing data for each variable of interest	10
Outcome data	15*	Report numbers of outcome events or summary measures	11, 12, 13 Figure 2, 3, 4
Main results	16	(*a*) Give unadjusted estimates and, if applicable, confounder-adjusted estimates and their precision (eg, 95% confidence interval). Make clear which confounders were adjusted for and why they were included	11, 12, 13 Appendix p.9, 10, 11
		(*b*) Report category boundaries when continuous variables were categorized	N/A
		(*c*) If relevant, consider translating estimates of relative risk into absolute risk for a meaningful time period	N/A
Other analyses	17	Report other analyses done—eg analyses of subgroups and interactions, and sensitivity analyses	11 Appendix p.12, 13
**Discussion**			
Key results	18	Summarise key results with reference to study objectives	13
Limitations	19	Discuss limitations of the study, taking into account sources of potential bias or imprecision. Discuss both direction and magnitude of any potential bias	15, 16, 17
Interpretation	20	Give a cautious overall interpretation of results considering objectives, limitations, multiplicity of analyses, results from similar studies, and other relevant evidence	14, 15
Generalisability	21	Discuss the generalisability (external validity) of the study results	17
**Other information**			
Funding	22	Give the source of funding and the role of the funders for the present study and, if applicable, for the original study on which the present article is based	20

*Give information separately for exposed and unexposed groups.

Table S3. Classifications of active physical comorbidities

All active physical comorbidities	Active physical comorbidities at risk of COVID-19 complications according to INSPQ [6]	Active physical comorbidities at risk of COVID-19 complications according to NACI [34]
Diabetes	Diabetes	Diabetes
Chronic heart disease	Chronic heart disease	Chronic heart disease
Chronic liver disease	Chronic liver disease	Chronic liver disease
Chronic renal disease	Chronic renal disease	Chronic renal disease
Alzheimer disease or any other dementia	Alzheimer disease or any other dementia	Alzheimer disease or any other dementia
Chronic lung disease	Chronic lung disease	
Cancer	Cancer	
Immunosuppressing condition	Immunosuppressing condition	
Neurologic disease	Neurologic disease	
Hematologic disease	Hematologic disease	
Thyroid disease	Hypothyroidism	
Arrhythmia	Arrhythmia	
Vascular disease	Vascular disease	
Hypertension	Hypertension	
Physical disability	Paralysis	
Chronic inflammatory disease		
Asplenia or hyposplenia		
Arthritis or arthrosis		
Dermatological disease		
Multisystemic disease		
Endocrine disease		
Digestive disease		
Lymphatic disease		
Back or spinal condition		
Other chronic pain		
Sensory disability		
Infectious disease		
Sexually transmitted and blood-borne infections		
Orthopedic condition		
Migraine		
Vertigo or Meniere's disease		

INSPQ: Institut national de santé publique du Québec
NACI: National Advisory Committee on Immunization

Table S4. Sample size calculations

A) Detectable differences* for different scenarios† of proportion of individuals with comorbidities, sample size, design effect, and mean number of contacts among individuals with comorbidities

		Design effect¥															
		1				2				5				10			
		Mean number of contacts among individuals with comorbidities															
		1		3		1		3		1		3		1		3	
% with comorbidities	Sample size	Diff	Power	Diff	Power	Diff	Power	Diff	Power	Diff	Power	Diff	Power	Diff	Power	Diff	Power
22%	400	0.4	82	0.7	87	0.6	82	1.0	85	1.1	83	1.6	81	1.7	80	2.0	68
22%	800	0.3	88	0.5	89	0.4	82	0.7	87	0.7	83	1.1	83	1.1	83	1.6	81
22%	1200	0.3	97	0.4	88	0.4	94	0.6	90	0.6	88	0.9	85	0.8	80	1.3	83
35%	100	0.8	86	1.2	83	1.2	84	1.7	80	2.0	76	2.0	53	2.0	47	2.0	30
35%	200	0.5	83	0.8	82	0.8	86	1.2	83	1.3	80	2.0	82	2.0	76	2.0	53

* Differences of up to 2.0 were considered in power calculations. When a power greater than 80% could not be achieved with the maximum value of 2.0, the power for that difference is presented.

† Scenarios with proportion of 22% with comorbidities and n=400, 800, 1200 represent sample sizes observed for all adults and adults aged 18 to 65 years, and scenarios with proportion of 35% with comorbidities and n=100, 200 represent sample sizes observed for adults aged over 65 years.

¥ The design effect represents the inflation in variance between the observed variance in our study compared to the theoretical variance of a Poisson distribution with simple random sampling.

B) Observed design effects for comparisons of contacts between individuals with and without comorbidities*

	Total contacts		Contacts at home with household members		Contacts with visitors at home and contacts in other locations	
	Pre-COVID	During pandemic	Pre-COVID	During pandemic	Pre-COVID	During pandemic
All adults	11.2	5.1 to 9.5	1.2	0.7 to 1.3	12.3	5.9 to 11.2
18-65 years old	19.1	7.7 to 12.4	1.5	0.7 to 1.1	20.9	9.1 to 15.1
>65 years old	2.9	2.0 to 9.9	0.9	0.6 to 2.1	3.5	3.2 to 10.1

* Based on comparisons of contacts presented in Table S6.

Table S5. Key socio-demographic characteristics of participants, by period

	Pre-COVID						1st wave						Summer 2020 and 2nd wave						3rd wave					
	All participants		With active physical comorbidities		Without active physical comorbidities		All participants		With active physical comorbidities		Without active physical comorbidities		All participants		With active physical comorbidities		Without active physical comorbidities		All participants		With active physical comorbidities		Without active physical comorbidities	
	N_{crude}	$\%_{weighted}$	N_{crude}	$\%_{weighted}$	N_{crude}	$\%_{weighted}$	N_{crude}	$\%_{weighted}$	N_{crude}	$\%_{weighted}$	N_{crude}	$\%_{weighted}$	N_{crude}	$\%_{weighted}$	N_{crude}	$\%_{weighted}$	N_{crude}	$\%_{weighted}$	N_{crude}	$\%_{weighted}$	N_{crude}	$\%_{weighted}$	N_{crude}	$\%_{weighted}$
**Total**	**792**		**203**		**589**		**413**		**100**		**313**		**4125**		**851**		**3274**		**1296**		**287**		**1009**	
**Age**																								
18-25 yrs old	95	10.9	5	1.4	90	14.1	53	11.8	8	5.6	45	14.2	532	12.0	38	3.6	494	14.2	185	11.7	18	4.5	167	13.8
26-45 yrs old	197	35.3	32	26.8	165	38.2	181	34.0	35	20.6	146	39.1	1369	34.4	193	21.6	1176	37.9	457	34.2	92	29.1	365	35.8
46-65 yrs old	294	33.0	87	39.4	207	30.8	129	32.8	37	34.6	92	32.1	1610	33.3	414	39.5	1196	31.6	470	32.5	106	32.2	364	32.6
66-75 yrs old	159	12.6	60	19.2	99	10.4	45	12.6	16	17.0	29	11.0	535	12.5	167	18.1	368	10.9	162	12.4	62	20.6	100	9.9
>75 yrs old	47	8.3	19	13.3	28	6.6	5	8.8	4	22.2	1	3.7	79	7.9	39	17.2	40	5.4	22	9.3	9	13.6	13	8.0
**Sex**																								
Male	350	50.1	91	49.4	259	50.3	169	49.1	39	49.7	130	48.9	2018	49.8	413	47.8	1605	50.4	577	50.2	127	51.3	450	49.9
Female	442	49.9	112	50.6	330	49.7	244	50.9	61	50.3	183	51.1	2107	50.2	438	52.2	1669	49.6	719	49.8	160	48.7	559	50.2
**Region**																								
Greater Montreal†	407	62.6	103	62.1	304	62.7	293	64.3	78	67.9	215	62.9	2356	61.2	486	62.8	1870	60.7	749	61.1	168	59.5	581	61.5
Other Quebec regions	377	37.5	99	37.9	278	37.3	120	35.8	22	32.1	98	37.2	1766	38.8	365	37.2	1401	39.3	545	38.9	119	40.5	426	38.5
Missing	8	--	1	--	7	--	--	--	--	--	--	--	3	--	--	--	3	--	2	--	--	--	2	--
**Household size**																								
1	236	30.0	72	35.6	164	28.2	124	29.9	32	28.3	92	30.5	1007	24.7	252	30.5	755	23.1	328	27.4	77	29.3	251	26.9
2	389	43.1	107	45.9	282	42.2	182	45.5	53	60.5	129	39.7	2086	49.3	473	55.7	1613	47.5	624	47.1	156	52.5	468	45.5
3	87	10.9	15	10.3	72	11.1	41	9.3	5	3.6	36	11.5	488	11.9	58	6.1	430	13.5	173	12.6	29	9.2	144	13.7
4+	80	16.0	9	8.2	71	18.5	66	15.4	10	7.6	56	18.4	544	14.2	68	7.7	476	15.9	171	12.9	25	9.1	146	14.0
**Education Level**																								
No diploma, degree	64	7.8	23	10.7	41	6.9	20	5.1	6	5.6	14	4.9	172	4.1	44	4.7	128	3.9	45	3.5	10	4.1	35	3.3
Secondary (high) school	120	14.4	28	15.1	92	14.1	56	14.5	14	13.6	42	14.8	561	13.9	118	13.8	443	14.0	153	11.0	32	9.8	121	11.4
College, CEGEP, university or other certificate/diploma	608	77.8	152	74.2	456	79.0	337	80.5	80	80.8	257	80.4	3392	82.0	689	81.5	2703	82.1	1098	85.5	245	86.2	853	85.3
**Main occupation**																								
Student employed or unemployed	76	10.8	6	3.2	70	13.3	51	10.4	9	5.2	42	12.5	481	11.1	47	4.9	434	12.8	153	10.3	22	7.2	131	11.2
Employed or semi-retired	363	49.5	61	33.7	302	54.8	230	50.6	53	40.7	177	54.4	2435	56.6	431	45.7	2004	59.5	766	55.7	143	44.4	623	59.1
Temporarily not working or seeking work	18	2.6	6	4.0	12	2.1	59	12.4	12	10.3	47	13.2	266	5.9	75	7.5	191	5.5	94	6.4	25	7.5	69	6.1
Unemployed or retired	335	37.1	130	59.2	205	29.8	73	26.6	26	43.8	47	19.9	943	26.5	298	41.9	645	22.2	283	27.7	97	40.9	186	23.7

	Pre-COVID						1st wave						Summer 2020 and 2nd wave						3rd wave					
	All participants		With active physical comorbidities		Without active physical comorbidities		All participants		With active physical comorbidities		Without active physical comorbidities		All participants		With active physical comorbidities		Without active physical comorbidities		All participants		With active physical comorbidities		Without active physical comorbidities	
	N_{crude}	$\%_{weighted}$	N_{crude}	$\%_{weighted}$	N_{crude}	$\%_{weighted}$	N_{crude}	$\%_{weighted}$	N_{crude}	$\%_{weighted}$	N_{crude}	$\%_{weighted}$	N_{crude}	$\%_{weighted}$	N_{crude}	$\%_{weighted}$	N_{crude}	$\%_{weighted}$	N_{crude}	$\%_{weighted}$	N_{crude}	$\%_{weighted}$	N_{crude}	$\%_{weighted}$
**Type of employment***																								
Education	54	13.5	9	10.0	45	14.1	27	9.1	8	13.0	19	7.9	228	8.5	40	8.6	188	8.5	90	10.0	18	11.5	72	9.6
Health	45	11.2	9	11.2	36	11.3	30	9.3	6	8.1	24	9.6	227	8.2	46	9.4	181	8.0	77	8.5	18	10.5	59	8.0
Sales and services	58	14.7	9	18.0	49	14.0	35	11.8	7	11.3	28	12.0	336	12.8	61	13.1	275	12.7	108	12.5	21	13.3	87	12.3
Other sectors	215	60.6	34	60.8	181	60.6	188	69.8	44	67.7	144	70.5	1841	70.5	318	68.9	1523	70.9	558	69.1	94	64.8	464	70.1
Missing	--	--	--	--	--	--	1	--	--	--	1	--	18	--	3	--	15	--	4	--	--	--	4	--
**Race/Ethnicity**																								
White	728	91.6	192	95.0	536	90.5	355	89.3	93	94.8	262	87.2	3750	91.7	796	95.0	2954	90.8	1150	89.9	256	89.6	894	90.0
Other	57	8.4	11	5.1	46	9.5	51	10.7	7	5.2	44	12.8	325	8.3	44	5.0	281	9.2	128	10.1	26	10.4	102	10.0
Missing	7	--	--	--	7	--	7	--	--	--	7	--	50	--	11	--	39	--	18	--	5	--	13	--
**Country of origin**																								
Canadian-born	729	90.4	195	94.9	534	88.9	348	86.8	91	94.5	257	83.9	3696	88.6	789	91.9	2907	87.7	1144	88.1	255	87.9	889	88.2
Foreign-born	63	9.6	8	5.1	55	11.1	64	13.2	9	5.5	55	16.1	422	11.4	62	8.1	360	12.3	151	11.9	32	12.1	119	11.8
Missing	--	--	--	--	--	--	1	--	--	--	1	--	7	--	--	--	7	--	1	--	--	--	1	--
**Number of active physical comorbidities**																								
1	--	--	143	71.2	--	--	--	--	79	74.6	--	--	--	--	672	79.6	--	--	--	--	234	82.0	--	--
2	--	--	44	22.2	--	--	--	--	14	18.8	--	--	--	--	143	15.7	--	--	--	--	45	15.6	--	--
3+	--	--	16	6.6	--	--	--	--	7	6.5	--	--	--	--	36	4.7	--	--	--	--	8	2.4	--	--
**Type of comorbidities**																								
Chronic lung disease	--	--	41	19.4	--	--	--	--	33	28.0	--	--	--	--	182	20.6	--	--	--	--	59	18.6	--	--
Diabetes	--	--	54	23.7	--	--	--	--	19	23.2	--	--	--	--	176	20.2	--	--	--	--	59	21.6	--	--
Chronic inflammatory disease	--	--	35	16.7	--	--	--	--	11	7.8	--	--	--	--	141	17.2	--	--	--	--	49	15.0	--	--
Hypertension	--	--	25	10.7	--	--	--	--	9	8.2	--	--	--	--	101	14.1	--	--	--	--	30	12.5	--	--
Chronic heart disease	--	--	29	14.6	--	--	--	--	3	6.0	--	--	--	--	81	9.6	--	--	--	--	24	10.2	--	--
Cancer	--	--	19	9.6	--	--	--	--	8	18.9	--	--	--	--	51	6.7	--	--	--	--	20	7.3	--	--
Thyroid disease	--	--	10	6.5	--	--	--	--	6	4.3	--	--	--	--	60	6.9	--	--	--	--	20	8.2	--	--
Neurologic disease	--	--	14	5.4	--	--	--	--	8	6.5	--	--	--	--	55	5.7	--	--	--	--	12	3.9	--	--
Arthritis and arthrosis	--	--	16	8.3	--	--	--	--	5	4.3	--	--	--	--	36	4.1	--	--	--	--	8	2.5	--	--
Others	--	--	36	19.5	--	--	--	--	24	22.7	--	--	--	--	177	19.5	--	--	--	--	66	20.4	--	--

Pre-COVID: February 1st 2018 to March 17th 2019; **1st wave:** April 21st to May 25th 2020; **Summer 2020 and 2nd wave:** July 3rd 2020 to March 20th 2021; **3rd wave:** March 21st to July 4th 2021.

† Greater Montreal: Regions of Montreal, Laval, Montérégie, Lanaudière, Laurentides

* Type of employment: among 18-65-year-olds employed or temporarily not working

Table S6. Time trends in the mean number of social contacts of individuals with and without active physical comorbidities

A) Total contacts

	Pre-COVID n=792		1st wave n=413			Summer 2020 n=679			2nd wave before holidays n=1261			Holidays n=1197			2nd wave after holidays n=988			3rd wave n=1296		
	**mean**	**95% CI**	**mean**	**95% CI**	***p***[¥]	**mean**	**95% CI**	***p***[¥]	**mean**	**95% CI**	***p***[¥]	**mean**	**95% CI**	***p***[¥]	**mean**	**95% CI**	***p***[¥]	**mean**	**95% CI**	***p***[¥]
**All adults**[*] **Total**	7.6	(6.9-8.3)	2.9	(2.5-3.3)	*<.001*	4.3	(3.7-4.8)	*<.001*	3.6	(3.3-3.9)	*0.05*	2.9	(2.6-3.1)	*<.001*	3.3	(3.0-3.6)	*0.02*	4.4	(4.0-4.7)	*<.001*
Without comorbidities	8.1	(7.3-9.0)	2.7	(2.2-3.2)	*<.001*	4.3	(3.6-5.0)	*<.001*	3.9	(3.5-4.3)	*0.38*	2.8	(2.5-3.1)	*<.001*	3.4	(3.0-3.7)	*0.01*	4.5	(4.1-4.9)	*<.001*
With comorbidities	6.1	(4.9-7.3)	3.2	(2.5-3.9)	*<.001*	4.2	(3.5-4.9)	*0.03*	2.9	(2.5-3.2)	*0.001*	3.0	(2.6-3.4)	*0.67*	3.0	(2.6-3.5)	*0.81*	4.1	(3.4-4.7)	*0.009*
Diff[†] *(p-value)*	-2.0	*(0.008)*	0.5	*(0.23)*		-0.1	*(0.92)*		-1.0	*(<.001)*		0.2	*(0.41)*		-0.3	*(0.30)*		-0.4	*(0.27)*	
**18-65 years old**[*] **Total**	8.9	(8.0-9.9)	3.3	(2.6-3.9)	*<.001*	4.4	(3.8-5.0)	*0.009*	4.2	(3.8-4.7)	*0.66*	3.1	(2.8-3.5)	*<.001*	3.7	(3.2-4.1)	*0.05*	5.1	(4.6-5.5)	*<.001*
Without comorbidities	9.3	(8.2-10.4)	3.2	(2.5-3.9)	*<.001*	4.3	(3.6-5.0)	*0.02*	4.5	(4.0-5.0)	*0.66*	3.1	(2.7-3.5)	*<.001*	3.9	(3.3-4.4)	*0.02*	5.2	(4.7-5.7)	*<.001*
With comorbidities	7.7	(5.7-9.7)	3.5	(1.9-5.0)	*0.001*	4.9	(3.6-6.3)	*0.16*	3.2	(2.6-3.8)	*0.03*	3.2	(2.4-4.0)	*0.91*	2.8	(2.1-3.6)	*0.51*	4.6	(3.6-5.6)	*0.006*
Diff[†] *(p-value)*	-1.6	*(0.16)*	0.3	*(0.76)*		0.7	*(0.40)*		-1.2	*(0.003)*		0.1	*(0.84)*		-1.0	*(0.03)*		-0.6	*(0.34)*	
**>65 years old Total**	3.5	(3.0-4.0)	1.2	(0.7-1.6)	*<.001*	2.8	(1.9-3.7)	*0.002*	1.5	(1.3-1.8)	*0.008*	1.2	(1.0-1.5)	*0.10*	1.5	(1.2-1.9)	*0.13*	2.1	(1.7-2.6)	*0.05*
Without comorbidities	4.0	(3.3-4.8)	1.0	(0.3-1.7)	*<.001*	3.3	(1.8-4.7)	*0.006*	1.8	(1.5-2.0)	*0.05*	1.3	(0.9-1.6)	*0.01*	1.5	(1.1-2.0)	*0.35*	2.1	(1.5-2.8)	*0.12*
With comorbidities	2.8	(2.3-3.3)	1.3	(0.8-1.8)	*<.001*	2.1	(1.4-2.7)	*0.07*	1.0	(0.6-1.4)	*0.009*	1.2	(0.9-1.5)	*0.46*	1.6	(1.1-2.1)	*0.18*	2.1	(1.4-2.8)	*0.22*
Diff[†] *(p-value)*	-1.2	*(0.006)*	0.3	*(0.49)*		-1.2	*(0.13)*		-0.8	*(<.001)*		-0.1	*(0.78)*		0.1	*(0.87)*		-0.0	*(0.93)*	

Pre-COVID: February 1st 2018 to March 17th 2019; **1st wave:** April 21st to May 25th 2020; **Summer 2020:** July 3rd to August 22nd 2020; **2nd wave:** August 23rd 2020 to March 20th 2021; **Holidays:** December 17th 2020 to January 8th 2021; **3rd wave:** March 21st to July 4th 2021.

[¥] p: p-value of the difference with the preceding period

[*] Results for all adults and 18-65-year-olds are adjusted for age

[†] Diff: Difference between individuals with and without comorbidities and p-value of this difference

B) Contacts at home with household members

	Pre-COVID n=792		1st wave n=413			Summer 2020 n=679			2nd wave before holidays n=1261			Holidays n=1197			2nd wave after holidays n=988			3rd wave n=1296		
	**mean**	**95% CI**	**mean**	**95% CI**	***p*[¥]**	**mean**	**95% CI**	***p*[¥]**	**mean**	**95% CI**	***p*[¥]**	**mean**	**95% CI**	***p*[¥]**	**mean**	**95% CI**	***p*[¥]**	**mean**	**95% CI**	***p*[¥]**
**All adults[*]** **Total**	0.9	(0.8-1.0)	0.9	(0.8-1.1)	*0.33*	0.9	(0.8-1.0)	*0.80*	1.0	(0.9-1.0)	*0.21*	1.0	(0.9-1.0)	*0.97*	1.0	(1.0-1.1)	*0.26*	1.0	(0.9-1.0)	*0.15*
Without comorbidities	0.9	(0.8-1.0)	0.9	(0.8-1.0)	*0.43*	0.9	(0.8-1.0)	*0.80*	1.0	(1.0-1.1)	*0.04*	1.0	(0.9-1.1)	*0.49*	1.0	(1.0-1.1)	*0.29*	1.0	(0.9-1.0)	*0.11*
With comorbidities	0.9	(0.8-1.0)	1.0	(0.7-1.2)	*0.57*	1.0	(0.8-1.1)	*0.99*	0.9	(0.8-1.0)	*0.31*	1.0	(0.9-1.1)	*0.25*	1.0	(0.9-1.1)	*0.79*	1.0	(0.9-1.1)	*0.98*
Diff[†] *(p-value)*	0.0	*(0.61)*	0.1	*(0.69)*		0.1	*(0.42)*		-0.1	*(0.03)*		-0.0	*(0.70)*		-0.1	*(0.40)*		0.0	*(0.72)*	
**18-65 years old[*]** **Total**	1.0	(0.9-1.1)	1.0	(0.9-1.1)	*0.63*	1.0	(0.9-1.1)	*0.71*	1.1	(1.0-1.1)	*0.10*	1.1	(1.0-1.2)	*0.87*	1.1	(1.1-1.2)	*0.36*	1.1	(1.1-1.2)	*0.58*
Without comorbidities	1.0	(0.9-1.1)	1.1	(1.0-1.2)	*0.24*	1.0	(0.9-1.1)	*0.23*	1.1	(1.0-1.2)	*0.08*	1.1	(1.0-1.2)	*0.93*	1.2	(1.1-1.2)	*0.30*	1.1	(1.0-1.2)	*0.30*
With comorbidities	1.0	(0.8-1.2)	0.8	(0.7-1.0)	*0.13*	1.0	(0.9-1.2)	*0.09*	1.0	(0.9-1.2)	*0.83*	1.1	(0.9-1.2)	*0.90*	1.0	(0.9-1.2)	*0.81*	1.1	(1.0-1.3)	*0.31*
Diff[†] *(p-value)*	0.0	*(0.96)*	-0.3	*(0.008)*		0.0	*(0.83)*		-0.1	*(0.38)*		-0.1	*(0.50)*		-0.1	*(0.09)*		0.0	*(0.76)*	
**>65 years old** **Total**	0.4	(0.3-0.5)	0.6	(0.3-0.8)	*0.37*	0.5	(0.4-0.7)	*0.87*	0.6	(0.5-0.7)	*0.69*	0.6	(0.5-0.7)	*0.86*	0.6	(0.5-0.7)	*0.48*	0.5	(0.4-0.6)	*0.08*
Without comorbidities	0.4	(0.3-0.5)	0.3	(0.1-0.5)	*0.58*	0.5	(0.3-0.8)	*0.25*	0.7	(0.6-0.8)	*0.21*	0.6	(0.5-0.7)	*0.20*	0.6	(0.5-0.7)	*0.78*	0.5	(0.3-0.6)	*0.18*
With comorbidities	0.5	(0.4-0.6)	0.8	(0.4-1.2)	*0.15*	0.6	(0.4-0.8)	*0.34*	0.4	(0.2-0.6)	*0.22*	0.6	(0.4-0.7)	*0.15*	0.6	(0.5-0.8)	*0.48*	0.5	(0.4-0.7)	*0.28*
Diff[†] *(p-value)*	0.1	*(0.32)*	0.5	*(0.04)*		0.1	*(0.71)*		-0.3	*(0.01)*		-0.0	*(0.99)*		0.0	*(0.65)*		0.1	*(0.59)*	

Pre-COVID: February 1st 2018 to March 17th 2019; **1st wave:** April 21st to May 25th 2020; **Summer 2020:** July 3rd to August 22nd 2020; **2nd wave:** August 23rd 2020 to March 20th 2021; **Holidays:** December 17th 2020 to January 8th 2021; **3rd wave:** March 21st to July 4th 2021.

[¥] p: p-value of the difference with the preceding period

[*] Results for all adults and 18-65-year-olds are adjusted for age

[†] Diff: Difference between individuals with and without comorbidities and p-value of this difference

C) Contacts with visitors at home and contacts in other locations

	Pre-COVID n=792		1st wave n=413			Summer 2020 n=679			2nd wave before holidays n=1261			Holidays n=1197			2nd wave after holidays n=988			3rd wave n=1296		
	**mean**	**95% CI**	**mean**	**95% CI**	***p*[¥]**	**mean**	**95% CI**	***p*[¥]**	**mean**	**95% CI**	***p*[¥]**	**mean**	**95% CI**	***p*[¥]**	**mean**	**95% CI**	***p*[¥]**	**mean**	**95% CI**	***p*[¥]**
**All adults[*]** **Total**	6.8	(6.1-7.5)	2.0	(1.7-2.3)	*<.001*	3.3	(2.8-3.8)	*<.001*	2.6	(2.3-2.9)	*0.02*	1.9	(1.7-2.1)	*<.001*	2.2	(2.0-2.5)	*0.05*	3.4	(3.1-3.8)	*<.001*
Without comorbidities	7.3	(6.4-8.2)	1.8	(1.4-2.2)	*<.001*	3.4	(2.7-4.0)	*<.001*	2.8	(2.5-3.2)	*0.17*	1.8	(1.6-2.1)	*<.001*	2.3	(1.9-2.6)	*0.03*	3.5	(3.1-3.9)	*<.001*
With comorbidities	5.3	(4.0-6.5)	2.1	(1.6-2.6)	*<.001*	3.2	(2.5-3.8)	*0.009*	2.0	(1.7-2.3)	*0.002*	2.0	(1.6-2.3)	*0.95*	2.1	(1.7-2.5)	*0.74*	3.1	(2.5-3.7)	*0.005*
Diff[†] *(p-value)*	-2.1	*(0.008)*	0.3	*(0.29)*		-0.2	*(0.68)*		-0.8	*(<.001)*		0.2	*(0.44)*		-0.2	*(0.40)*		-0.5	*(0.23)*	
**18-65 years old[*]** **Total**	8.0	(7.0-8.9)	2.2	(1.6-2.8)	*<.001*	3.4	(2.8-4.0)	*0.006*	3.1	(2.7-3.6)	*0.42*	2.0	(1.7-2.4)	*<.001*	2.5	(2.1-3.0)	*0.06*	4.0	(3.5-4.4)	*<.001*
Without comorbidities	8.3	(7.2-9.4)	2.1	(1.4-2.8)	*<.001*	3.3	(2.7-4.0)	*0.01*	3.3	(2.8-3.8)	*0.99*	2.0	(1.6-2.4)	*<.001*	2.7	(2.2-3.2)	*0.03*	4.1	(3.5-4.6)	*<.001*
With comorbidities	6.7	(4.7-8.7)	2.7	(1.2-4.2)	*0.001*	3.9	(2.5-5.3)	*0.23*	2.2	(1.6-2.8)	*0.03*	2.1	(1.3-2.9)	*0.89*	1.8	(1.1-2.6)	*0.56*	3.5	(2.5-4.5)	*0.007*
Diff[†] *(p-value)*	-1.6	*(0.17)*	0.6	*(0.51)*		0.6	*(0.47)*		-1.1	*(0.004)*		0.2	*(0.74)*		-0.9	*(0.06)*		-0.6	*(0.33)*	
**>65 years old** **Total**	3.1	(2.6-3.6)	0.6	(0.3-0.9)	*<.001*	2.2	(1.4-3.0)	*<.001*	0.9	(0.7-1.2)	*0.003*	0.7	(0.4-0.9)	*0.09*	0.9	(0.6-1.2)	*0.17*	1.6	(1.2-2.1)	*0.01*
Without comorbidities	3.6	(2.9-4.4)	0.7	(0.1-1.2)	*<.001*	2.8	(1.5-4.0)	*0.003*	1.1	(0.8-1.4)	*0.01*	0.7	(0.4-1.0)	*0.05*	0.9	(0.5-1.4)	*0.37*	1.7	(1.1-2.3)	*0.05*
With comorbidities	2.3	(1.8-2.8)	0.5	(0.3-0.8)	*<.001*	1.5	(0.8-2.1)	*0.006*	0.6	(0.3-0.9)	*0.02*	0.6	(0.4-0.9)	*0.98*	0.9	(0.5-1.4)	*0.22*	1.6	(0.9-2.2)	*0.11*
Diff[†] *(p-value)*	-1.3	*(0.004)*	-0.2	*(0.59)*		-1.3	*(0.07)*		-0.5	*(0.02)*		-0.1	*(0.76)*		0.0	*(0.97)*		-0.1	*(0.83)*	

Pre-COVID: February 1st 2018 to March 17th 2019; **1st wave:** April 21st to May 25th 2020; **Summer 2020:** July 3rd to August 22nd 2020; **2nd wave:** August 23rd 2020 to March 20th 2021; **Holidays:** December 17th 2020 to January 8th 2021; **3rd wave:** March 21st to July 4th 2021.

[¥] p: p-value of the difference with the preceding period

[*] Results for all adults and 18-65-year-olds are adjusted for age

[†] Diff: Difference between individuals with and without comorbidities and p-value of this difference

Table S7. Time trends in the mean total number of social contacts of individuals with and without active physical comorbidities at risk of COVID-19 complications

A) According to INSPQ

	Pre-COVID n=792		1st wave n=413			Summer 2020 n=679			2nd wave before holidays n=1261			Holidays n=1197			2nd wave after holidays n=988			3rd wave n=1296		
	**mean**	**95% CI**	**mean**	**95% CI**	***p*[¥]**	**mean**	**95% CI**	***p*[¥]**	**mean**	**95% CI**	***p*[¥]**	**mean**	**95% CI**	***p*[¥]**	**mean**	**95% CI**	***p*[¥]**	**mean**	**95% CI**	***p*[¥]**
**All adults[*]**																				
Without comorbidities	7.9	(7.1-8.7)	2.8	(2.3-3.3)	*<.001*	4.4	(3.7-5.0)	*<.001*	3.8	(3.4-4.1)	*0.12*	2.9	(2.6-3.1)	*<.001*	3.3	(3.0-3.7)	*0.04*	4.4	(4.0-4.8)	*<.001*
With comorbidities	6.3	(4.7-7.8)	3.0	(2.4-3.7)	*<.001*	3.7	(3.1-4.3)	*0.11*	3.0	(2.6-3.4)	*0.05*	2.8	(2.4-3.1)	*0.44*	3.1	(2.6-3.7)	*0.29*	4.1	(3.4-4.9)	*0.02*
Diff[†] *(p-value)*	-1.6	*(0.06)*	0.2	*(0.68)*		-0.7	*(0.14)*		-0.8	*(0.002)*		-0.1	*(0.74)*		-0.2	*(0.55)*		-0.3	*(0.47)*	
**18-65 years old[*]**																				
Without comorbidities	9.1	(8.1-10.1)	3.4	(2.7-4.1)	*<.001*	4.5	(3.8-5.2)	*0.03*	4.4	(3.9-4.9)	*0.79*	3.2	(2.8-3.6)	*<.001*	3.8	(3.3-4.3)	*0.07*	5.1	(4.6-5.6)	*<.001*
With comorbidities	8.0	(5.3-10.7)	2.6	(1.1-4.1)	*0.001*	3.9	(3.0-4.9)	*0.13*	3.3	(2.6-4.1)	*0.32*	2.6	(1.9-3.2)	*0.10*	2.9	(2.0-3.9)	*0.51*	4.8	(3.5-6.1)	*0.02*
Diff[†] *(p-value)*	-1.0	*(0.48)*	-0.8	*(0.32)*		-0.6	*(0.35)*		-1.0	*(0.02)*		-0.6	*(0.09)*		-0.8	*(0.12)*		-0.3	*(0.68)*	
**>65 years old**																				
Without comorbidities	3.8	(3.2-4.5)	1.0	(0.3-1.7)	*<.001*	3.2	(2.0-4.5)	*0.003*	1.7	(1.4-1.9)	*0.02*	1.3	(1.0-1.6)	*0.06*	1.5	(1.1-1.9)	*0.33*	2.1	(1.5-2.8)	*0.10*
With comorbidities	2.9	(2.4-3.4)	1.3	(0.8-1.8)	*<.001*	1.8	(1.1-2.5)	*0.28*	1.1	(0.7-1.5)	*0.11*	1.2	(0.8-1.5)	*0.86*	1.6	(1.0-2.2)	*0.19*	2.1	(1.4-2.8)	*0.30*
Diff[†] *(p-value)*	-1.0	*(0.02)*	0.3	*(0.47)*		-1.4	*(0.05)*		-0.6	*(0.03)*		-0.1	*(0.65)*		0.1	*(0.84)*		-0.1	*(0.90)*	

INSPQ: Institut national de santé publique du Québec

Pre-COVID: February 1st 2018 to March 17th 2019; **1st wave:** April 21st to May 25th 2020; **Summer 2020:** July 3rd to August 22nd 2020; **2nd wave:** August 23rd 2020 to March 20th 2021; **Holidays:** December 17th 2020 to January 8th 2021; **3rd wave:** March 21st to July 4th 2021.

[¥] p: p-value of the difference with the preceding period

[*] Results for all adults and 18-65-year-olds are adjusted for age

[†] Diff: Difference between individuals with and without comorbidities and p-value of this difference

B) According to NACI

	Pre-COVID n=792		1st wave n=413			Summer 2020 n=679			2nd wave before holidays n=1261			Holidays n=1197			2nd wave after holidays n=988			3rd wave n=1296		
	**mean**	**95% CI**	**mean**	**95% CI**	***p*[¥]**	**mean**	**95% CI**	***p*[¥]**	**mean**	**95% CI**	***p*[¥]**	**mean**	**95% CI**	***p*[¥]**	**mean**	**95% CI**	***p*[¥]**	**mean**	**95% CI**	***p*[¥]**
**All adults[*]**																				
Without comorbidities	7.9	(7.1-8.7)	2.9	(2.5-3.4)	*<.001*	4.3	(3.8-4.9)	*<.001*	3.7	(3.4-4.0)	*0.06*	2.8	(2.6-3.1)	*<.001*	3.3	(3.0-3.6)	*0.01*	4.4	(4.0-4.8)	*<.001*
With comorbidities	5.1	(4.1-6.1)	2.7	(2.2-3.2)	*<.001*	3.1	(2.4-3.7)	*0.41*	2.8	(2.3-3.2)	*0.46*	3.0	(2.5-3.4)	*0.55*	2.8	(2.1-3.5)	*0.75*	4.1	(3.0-5.1)	*0.05*
Diff[†] *(p-value)*	-2.8	*(<.001)*	-0.2	*(0.52)*		-1.3	*(0.004)*		-0.9	*(0.001)*		0.1	*(0.61)*		-0.5	*(0.19)*		-0.4	*(0.54)*	
**18-65 years old[*]**																				
Without comorbidities	9.1	(8.1-10.1)	3.4	(2.7-4.0)	*<.001*	4.5	(3.8-5.1)	*0.02*	4.3	(3.9-4.8)	*0.72*	3.1	(2.8-3.5)	*<.001*	3.8	(3.3-4.2)	*0.03*	5.1	(4.6-5.5)	*<.001*
With comorbidities	5.9	(3.8-8.1)	1.2	(0.9-1.6)	*<.001*	3.4	(2.0-4.9)	*0.003*	2.6	(1.9-3.4)	*0.33*	2.8	(2.0-3.7)	*0.75*	2.0	(1.5-2.6)	*0.12*	5.3	(2.6-8.0)	*0.02*
Diff[†] *(p-value)*	-3.2	*(0.008)*	-2.1	*(<.001)*		-1.0	*(0.19)*		-1.7	*(<.001)*		-0.3	*(0.50)*		-1.7	*(<.001)*		0.3	*(0.85)*	
**>65 years old**																				
Without comorbidities	3.7	(3.1-4.3)	1.2	(0.7-1.8)	*<.001*	3.0	(2.0-4.0)	*0.003*	1.5	(1.3-1.8)	*0.007*	1.3	(1.0-1.5)	*0.16*	1.6	(1.2-2.0)	*0.22*	2.2	(1.7-2.8)	*0.06*
With comorbidities	3.0	(2.3-3.6)	1.0	(0.4-1.7)	*<.001*	1.4	(0.5-2.3)	*0.52*	1.3	(0.6-2.0)	*0.86*	1.1	(0.7-1.5)	*0.62*	1.4	(1.0-1.9)	*0.32*	1.7	(1.0-2.4)	*0.45*
Diff[†] *(p-value)*	-0.7	*(0.10)*	-0.2	*(0.69)*		-1.6	*(0.02)*		-0.2	*(0.53)*		-0.2	*(0.52)*		-0.2	*(0.62)*		-0.5	*(0.30)*	

NACI: National Advisory Committee on Immunization

Pre-COVID: February 1st 2018 to March 17th 2019; **1st wave:** April 21st to May 25th 2020; **Summer 2020:** July 3rd to August 22nd 2020; **2nd wave:** August 23rd 2020 to March 20th 2021; **Holidays:** December 17th 2020 to January 8th 2021; **3rd wave:** March 21st to July 4th 2021.

[¥] p: p-value of the difference with the preceding period

[*] Results for all adults and 18-65-year-olds are adjusted for age

[†] Diff: Difference between individuals with and without comorbidities and p-value of this difference

Table S8. Vaccination coverage with one dose of individuals with and without active physical comorbidities in the third wave

	18-25 yrs old n=199		26-45 yrs old n=492		46-65 yrs old n=511		>65 yrs old n=192	
	**mean (%)**	**95% CI**	**mean (%)**	**95% CI**	**mean (%)**	**95% CI**	**mean (%)**	**95% CI**
Without comorbidities	44.0	(36.6-51.4)	52.0	(46.7-57.1)	67.6	(62.9-72.3)	89.1	(80.1-98.0)
With comorbidities	25.4	(3.8-47.0)	57.9	(47.7-68.2)	66.5	(57.7-75.2)	93.8	(89.0-98.5)
Diff† *(p-value)*	-18.6	*(0.11)*	6.0	*(0.31)*	-1.1	*(0.83)*	4.7	*(0.36)*

Third wave: March 21st to July 4th 2021.

† Diff: Difference between individuals with and without comorbidities and p-value of this difference

Table S9. Recommended Quebec priority groups for vaccination against COVID-19 [14]

Priority order	Groups
**1**	Elderly people living in long-term care facilities (nursing homes, CHSLD)
**2**	Health care workers
**3**	Elderly people living in retirement homes
**4**	Isolated and remote communities
**5**	Adults aged over 80 years
**6**	Adults aged 70 to 79 years
**7**	Adults aged 60 to 69 years
**8**	Adults aged under 60 years with comorbidities at risk of COVID-19 complications*
**9**	Adults aged under 60 years without comorbidities working in essential services
**10**	General adult population
**11**	Children

* In practice, only individuals aged under 60 years with conditions at very high risk of COVID-19 complications (hospitalised patients with comorbidities or outpatients with severe immunosuppressive conditions) were invited to be vaccinated before other individuals in their age group [48].

Table S10. Mean total number of social contacts in the third wave of individuals with and without active physical comorbidities according to vaccination status with one dose

	18-25 yrs old n=199			26-45 yrs old n=492			46-65 yrs old n=511			>65 yrs old n=192		
	**n**	**mean**	**95% CI**	**n**	**mean**	**95% CI**	**n**	**mean**	**95% CI**	**n**	**mean**	**95% CI**
**Not vaccinated**												
Without comorbidities	101	4.9	(2.8-7.0)	189	4.6	(3.3-5.9)	129	3.3	(1.8-4.7)	11	2.8	(0.9-4.6)
With comorbidities	14	10.7	(4.9-16.5)	41	2.9	(1.7-4.1)	40	1.7	(0.0-3.8)	7	2.0	(1.2-2.8)
**Vaccinated**												
Without comorbidities	80	7.5	(4.6-10.5)	206	6.9	(5.4-8.4)	264	4.8	(3.8-5.9)	108	2.1	(1.5-2.7)
With comorbidities	4	6.0	(0.7-11.2)	56	5.9	(3.7-8.0)	78	5.2	(2.9-7.5)	66	2.1	(1.4-2.7)
**Between group differences**												
**Not vaccinated:** with – without comorbidities *(p-value)*		5.8	*(0.05)*		-1.7	*(0.03)*		-1.6	*(0.10)*		-0.7	*(0.46)*
**Vaccinated:** with – without comorbidities *(p-value)*		-1.6	*(0.60)*		-1.0	*(0.42)*		0.3	*(0.78)*		-0.0	*(0.98)*
**Without comorbidities:** vaccinated – not vaccinated *(p-value)*		2.6	*(0.21)*		2.2	*(0.05)*		1.6	*(0.13)*		-0.7	*(0.47)*
**With comorbidities:** vaccinated – not vaccinated *(p-value)*		-4.7	*(0.25)*		3.0	*(0.02)*		3.5	*(0.06)*		0.1	*(0.92)*

Third wave: March 21st to July 4th 2021.

Results are adjusted for the time periods (March, April, May, June-July).

Figure S1. Distribution of participants according to classifications of comorbidities

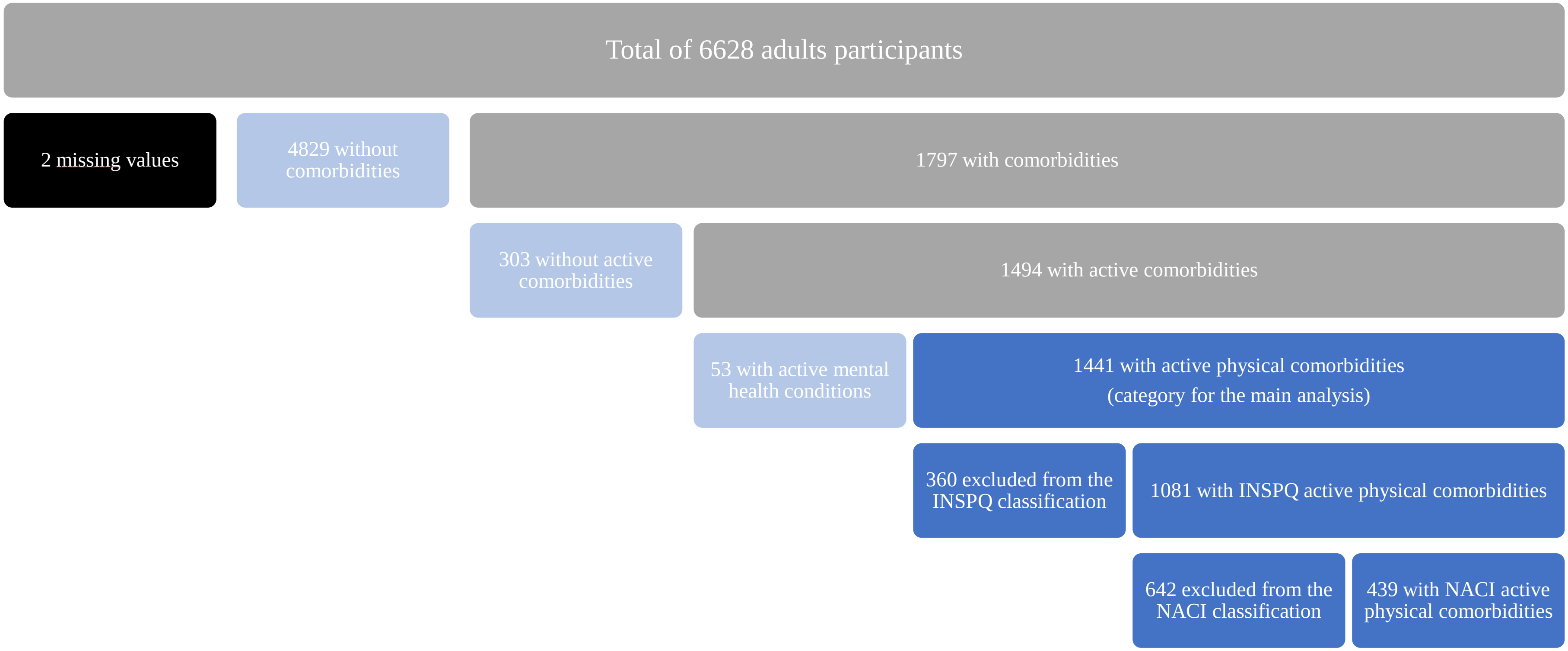

INSPQ: Institut national de santé publique du Québec
NACI: National Advisory Committee on Immunization

Figure S2. Time trends in vaccination coverage with one dose of individuals with and without active physical comorbidities in the third wave

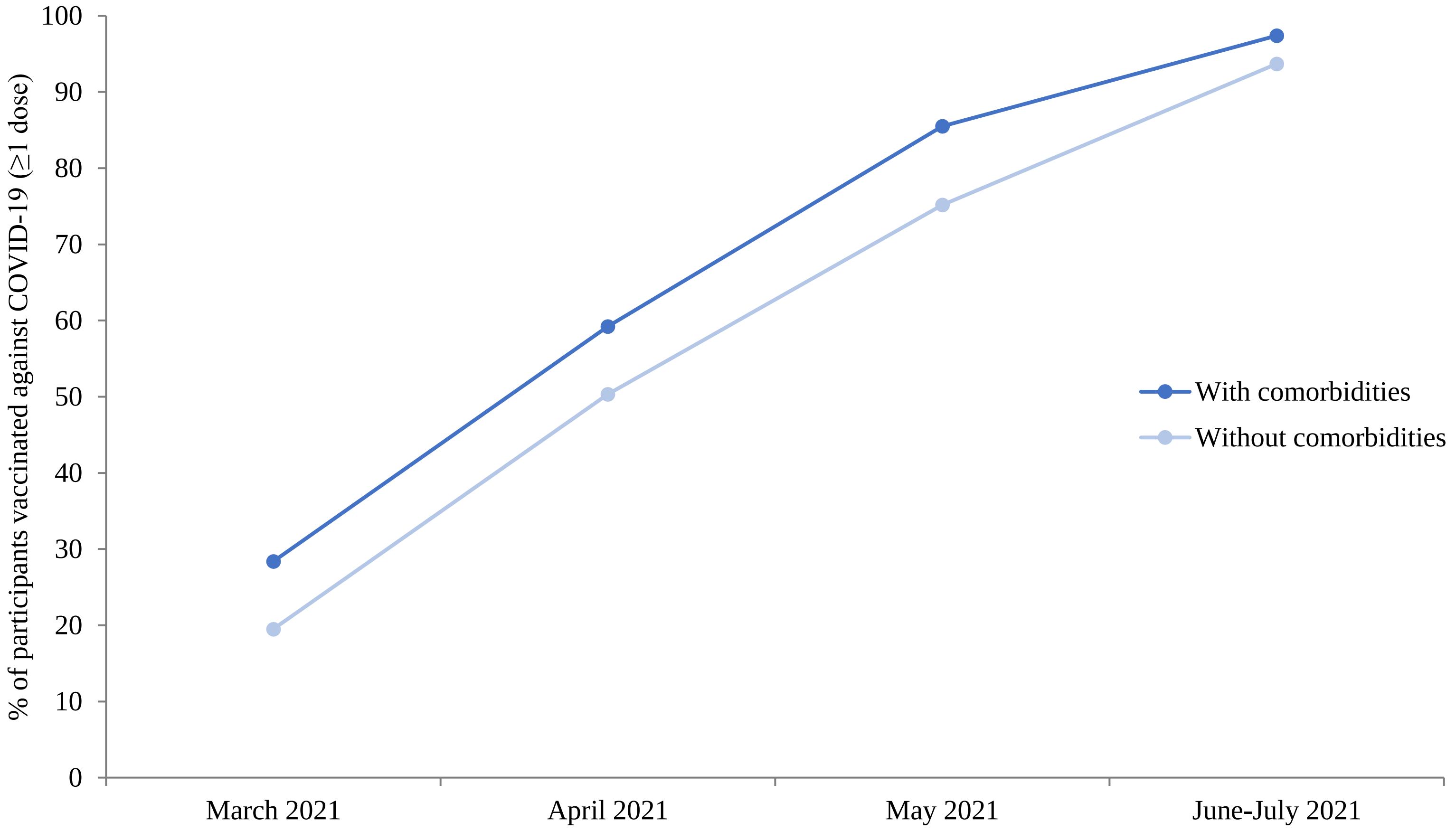

Third wave: March 21st to July 4th 2021.

Example of questions S1. Questions on health conditions

27. A) Which of the following long-term conditions do you have or have had?
(check all that apply)

Health conditions

- ☐ Chronic lung disease (e.g., asthma, chronic bronchitis, emphysema, chronic obstructive pulmonary disease (COPD), cystic fibrosis)
- ☐ Diabetes
- ☐ Chronic heart disease
- ☐ Chronic liver disease
- ☐ Chronic inflammatory disease (e.g., lupus erythematosus, rheumatoid arthritis, psoriasis, eczema, Crohn's disease, ulcerative colitis)
- ☐ Cancer
- ☐ Immunosuppressing condition (e.g., organ transplantation, HIV-infection, cancer chemotherapy, radiotherapy, congenital immunodeficiency)
- ☐ Chronic renal disease and/or dialysis
- ☐ Asplenia or hyposplenia (absent or defective spleen)
- ☐ Neurologic problem (e.g., Guillain-Barré syndrome, multiple sclerosis)
- ☐ Hematologic problem (e.g., anemia, hemoglobinopathy, bleeding problems)
- ☐ Alzheimer disease or any other dementia
- ☐ Another long-term health condition, specify: ____________________

27. B) For which of the following conditions have you had any symptoms or taken any medicine **in the past 12 months** ?

Health conditions

- ☐ Chronic lung disease (e.g., asthma, chronic bronchitis, emphysema, chronic obstructive pulmonary disease (COPD), cystic fibrosis)
- ☐ Diabetes
- ☐ Chronic heart disease
- ☐ Chronic liver disease
- ☐ Chronic inflammatory disease (e.g., lupus erythematosus, rheumatoid arthritis, psoriasis, eczema, Crohn's disease, ulcerative colitis)
- ☐ Cancer
- ☐ Immunosuppressing condition (e.g., organ transplantation, HIV-infection, cancer chemotherapy, radiotherapy, congenital immunodeficiency)
- ☐ Chronic renal disease and/or dialysis
- ☐ Asplenia or hyposplenia (absent or defective spleen)
- ☐ Neurologic problem (e.g., Guillain-Barré syndrome, multiple sclerosis)
- ☐ Hematologic problem (e.g., anemia, hemoglobinopathy, bleeding problems)
- ☐ Alzheimer disease or any other dementia
- ☐ Another long-term health condition, specify: ______________________
- ☐ No symptoms and no medicine for these conditions in the past 12 months

Example of questions S2. Example of the social contact diary

DATE DAY 1

☐☐☐☐	☐☐	☐☐
Year	Month	Day

List of persons with whom you have been in contact during this first day, from 5 am today to 5 am tomorrow morning.

Ini-tials or nick-name	Age (or age group) of the con-tacted person	Sex		Person's relation with yourself				Place of contact (*check all that apply*)						Total duration of contact with the person					Did you touch his/her skin?		How often do you have contact with this person in general?					Ethnicity of this person					
		Fe-male	Male	House-hold member	Family member not living in house-hold	Friend / Col-league	Other	Home / Car / Private place	Work	Kinder-garden / School / College / Univer-sity	Public trans-port	Lei-sure	Other	less than 5 min	5 - 14 min	15 - 59 min	1h - 4h	more than 4 h	Yes	No	Daily or almost daily	A few times a week	A few times a month	A few times a year or less	First time	White	Black	Asian	His-panic / Latino	Other	Don't know
____	☐☐(-☐☐)	☐	☐	☐	☐	☐	☐	☐	☐	☐	☐	☐	☐	☐	☐	☐	☐	☐	☐	☐	☐	☐	☐	☐	☐	☐	☐	☐	☐	☐	☐
____	☐☐(-☐☐)	☐	☐	☐	☐	☐	☐	☐	☐	☐	☐	☐	☐	☐	☐	☐	☐	☐	☐	☐	☐	☐	☐	☐	☐	☐	☐	☐	☐	☐	☐
____	☐☐(-☐☐)	☐	☐	☐	☐	☐	☐	☐	☐	☐	☐	☐	☐	☐	☐	☐	☐	☐	☐	☐	☐	☐	☐	☐	☐	☐	☐	☐	☐	☐	☐
____	☐☐(-☐☐)	☐	☐	☐	☐	☐	☐	☐	☐	☐	☐	☐	☐	☐	☐	☐	☐	☐	☐	☐	☐	☐	☐	☐	☐	☐	☐	☐	☐	☐	☐
____	☐☐(-☐☐)	☐	☐	☐	☐	☐	☐	☐	☐	☐	☐	☐	☐	☐	☐	☐	☐	☐	☐	☐	☐	☐	☐	☐	☐	☐	☐	☐	☐	☐	☐
____	☐☐(-☐☐)	☐	☐	☐	☐	☐	☐	☐	☐	☐	☐	☐	☐	☐	☐	☐	☐	☐	☐	☐	☐	☐	☐	☐	☐	☐	☐	☐	☐	☐	☐
